# Congenital Thrombotic Thrombocytopenic Purpura With a Novel ADAMTS13 Gene Mutation

**DOI:** 10.7759/cureus.12053

**Published:** 2020-12-13

**Authors:** Anila Rashid, Naureen Mushtaq, Huma Mansoori

**Affiliations:** 1 Department of Pathology and Laboratory Medicine, Aga Khan University Hospital, Karachi, PAK; 2 Department of Oncology, Aga Khan University Hospital, Karachi, PAK; 3 Department of Hematology, Dow University of Health Sciences, Karachi, PAK

**Keywords:** adamts13, hemolysis, thrombotic thrombocytopenic purpura

## Abstract

Congenital thrombotic thrombocytopenic purpura (TTP) is an autosomal recessive disorder characterized by thrombocytopenia, microangiopathic hemolytic anemia (MAHA), and thrombosis. Congenital TTP should also be considered while investigating neonatal hyperbilirubinemia, hemolytic anemia, or isolated thrombocytopenia. This case is of an 8-year-old male child who presented with prolonged and recurrent history of thrombocytopenia and MAHA, first identified when he was seven weeks of age preceding neonatal hyperbilirubinemia. Peripheral blood smear examination showed thrombocytopenia and schistocytes. He then went through a series of laboratory investigations until at the age of seven years, when the ADAMTS13 (a disintegrin and metalloproteinase with a thrombospondin type 1 motif, member 13) antigen level was performed and found to be low: 40 ng/ml (630-850). Subsequently, he received a trial of steroids and rituximab which were found to be ineffective and associated with complications. In this case, a definitive diagnosis was delayed until the age of eight years when a novel homozygous pathogenic frameshift variant ADAMTS13 c.3033delC, p.Cys1012AlafsX109 in exon 23 was identified. After receiving regular plasma infusions, thrombocytopenia and hemolysis improved. Congenital TTP should be considered in every neonatal hyperbilirubinemia, thrombocytopenia or hemolytic anemia to avoid delay in diagnosis. Early diagnosis through analysis of the ADAMTS13 gene is crucial for optimal management as well as for genetic counselling.

## Introduction

Upshaw-Schulman syndrome is a rare form of congenital thrombotic thrombocytopenic purpura (TTP) which results from loss of von Willebrand factor (VWF) cleaving protein, known as ADAMTS13 (a disintegrin and metalloproteinase with a thrombospondin type 1 motif, member 13) [1,2]. In the absence of ADAMTS13, ultra large multimers of VWF are not cleaved appropriately and leads to the formation of large aggregates of platelet under high shear conditions, including microvasculature of the kidneys, brain, and heart. The first ever case of congenital TTP was identified by Schulman et al. in 1960, in an 8-year-old girl who presented with low platelet count and microangiopathic hemolytic anemia (MAHA) [3].

Congenital TTP presents with MAHA, low platelet count, renal impairment, fluctuating neurological symptoms, and fever, often with subtle onset which may occur in early infancy [4]. Diagnosis can be intricate and sometimes delayed, as there are overlapping clinical features with autoimmune diseases, certain pregnancy-related complications, and hemolytic uremic syndrome [5]. Hyperbilirubinemia is a typical presentation of congenital TTP as a consequence of hemolysis warranting urgent exchange transfusion therapy to prevent kernicterus [6,7]. Alternatively, it can also present as isolated thrombocytopenia as an initial manifestation [8].

ADAMTS13 is a 37 kb gene located on chromosome 9q34 [9]. A large number of mutations have been identified along the entire coding sequence of the ADAMTS13 gene but none appears to predominate [10]. Hence the diagnosis of congenital TTP can only be confirmed through gene sequencing. Two cases of congenital TTP have been described from Pakistan [11,12]. In this report, we present a case of congenital TTP diagnosed with a homozygous frameshift variant at the age of eight years despite having clinical presentation in early infancy.

## Case presentation

An 8-year-old male, at our institute, was continuously being followed up for recurrent anemia and thrombocytopenia. He was born full term and presented with hyperbilirubinemia within 24 hours of birth but did not require exchange transfusion. He was then admitted at the age of seven weeks with fever. Laboratory evaluation showed thrombocytopenia and MAHA with the presence of numerous schistocytes on peripheral blood smear (Table 1).

**Table 1:** Laboratory workup in patient with congenital TTP TTP: thrombotic thrombocytopenic purpura; APTT: activated partial thromboplastin time; DAT: direct antiglobulin test; LDH: lactate dehydrogenase; BUN: blood urea nitrogen

Laboratory tests	Results									
Age	7 weeks	1 year	2 year	3 year	4 year	5 year	6 year	7 year	8 year	9 year
Hemoglobin gm/dl (Reference range)	7.4 (11.5-16.5)	7.7 (11.1-14.1)	8.4 (11.0-14)	5.8 (11.0-14)	7.6 (11.0-14)	8 (11.0-14)	10 (11.0-14)	12.1 (11.5-15.5)	13.1 (11.5-15.5)	13.2 (11.5-15.5)
Platelets 10^9^/L (Reference range)	27 (150-400)	15	11	26	19	24	43	96	102	148
WBC (10^9^/L)	8.1 (4-10)									
Reticulocyte count (%)	5.4% (0.8-2.1)									
PT seconds	10.1 (9.1-13.1)									
APTT seconds	28.7 (22.9-34.5)									
DAT	Negative									
LDH (IU/L)	1383 (208-378)									
Serum total bilirubin (mg/dL)	24.3 (0.2-1.2)									
Serum indirect bilirubin (mg/dL)	23.7									
BUN (mg/dL)	23 (6-20)									
Serum creatinine (mg/dL)	0.3 (0.3-0.7)									

His blood culture grew Streptococcus pneumonia. He was treated for infection-induced hemolytic anemia, however, bicytopenia (anemia and thrombocytopenia) persisted. At the age of six months, bone marrow aspirate and trephine were performed that exhibited erythroid hyperplasia with adequate megakaryopoiesis consistent with MAHA. Since that time, he has had several episodes of bicytopenia although he remained otherwise healthy and asymptomatic. There were no delayed milestones and no reported neurological symptoms. He has had no episodes of seizures and is in an appropriate school grade for his age. Family history is notable for non-consanguineous marriage and no family history of any haematological disorder.

The hematological picture, particularly thrombocytopenia did not improve despite a trial of steroids at the age of six year. He was followed monthly with a complete blood count and intermittently required platelet and packed red cell transfusion. Subsequently, ADAMTS13 antigen was tested and was found to be low: 40 ng/ml (630-850). Due to the non-availability of the ADAMTS13 inhibitor assay at our institute, a trial of immune modulation was attempted with four weekly doses of rituximab 375mg/m2 at the age of seven years which was complicated by hypertension, hematuria, proteinuria, and bicytopenia. Consequently, ADAMTS13 gene sequence analysis of the patient and his parents were performed at the Blood Center of Wisconsin, Milwaukee. The comparative analysis was done of the entire coding region and splice junction of each of 29 exons with the archived reference sequence and the functional implication of sequence variation was characterized.

A frameshift variant ADAMTS13 c.3033delC, p.Cys1012AlafsX109 in exon 23 was identified in both the alleles thus establishing the definitive diagnosis of congenital TTP. Both parents were found to be heterozygous for the same frameshift variant (Table 2).

**Table 2:** ADAMTS13 gene next-generation sequencing analysis

	Gene Transcript	Exon	DNA Change	Amino acid Change	Zygosity	Pathogenicity	Genomic Coordinates
Patient	NM_139025.4	Exon 23	c.3033delC	p.Cys1012AlafsX109	homozygous	pathogenic	Chr 9: g.136315075delC
Mother	NM_139025.4	Exon 23	c.3033delC	p.Cys1012AlafsX109	heterozygous	pathogenic	Chr 9: g.136315075delC
Father	NM_139025.4	Exon 23	c.3033delC	p.Cys1012AlafsX109	heterozygous	pathogenic	Chr 9: g.136315075delC

He was treated with fortnightly plasma infusions. This improved thrombocytopenia and hemolysis. On his last follow-up, the patient was kept on regular plasma infusions; had no further episodes of hemolysis, and cytopenias improved.

## Discussion

Congenital TTP, also known as Upshaw-Schulman syndrome, is a rare disorder caused by the deficiency of ADAMTS13 that presents with MAHA. Here, we have presented a case of a male child who had congenital TTP with persistent anemia and thrombocytopenia without systemic complications. This case is clinically significant as he had prolonged thrombocytopenia and hemolysis and underwent repeated irrelevant laboratory investigations like bone marrow and immune modulation treatment. The diagnosis was delayed until eight years of age when he was confirmed for congenital TTP on the basis of homozygous ADAMTS13 frameshift variant.

This patient followed a characteristic clinical course. After the first acute episode, most patients tend to present with a relapsing course [13]. Clinically it is characterized by single or recurrent bouts of thrombocytopenia, MAHA, and microvascular thrombosis. Other systemic complication usually involves cerebrovascular and renal dysfunction. Fortunately, our patient did not exhibit any such complications apart from thrombocytopenia and anemia until his last follow-up. TTP was once diagnosed based on the pentad of thrombocytopenia, MAHA, fever, neurologic and renal abnormalities [14]. However, with an improved understanding of this condition, the presence of otherwise unexplained thrombocytopenia and MAHA along with low ADAMTS13 activity (less than 10%) is sufficient to establish a diagnosis and begin therapy.

More than seventy ADAMTS13 mutations have been reported since 2001 including deletions, nonsense mutations, missense mutations, splice-site mutations, and insertions [15]. Approximately three fourth of all reported congenital TTP cases were compound heterozygous (64%), while almost a third of patients were homozygous (36%) for ADAMTS13 mutations [16]. The proteomic effect of mutations can be determined by the availability of tools like Genome Aggregation Database (gnomAD), Sorting Intolerant From Tolerant (SIFT), and Polymorphism Phenotyping version 2 (Polyphen-2) which can harmonize exome and genome sequencing data and assess the impact of a mutation on function.

The frameshift variant ADAMTS13 c.3033delC, p.Cys1012AlafsX109 (p. C1012AfsX109) in exon 23 changes the amino acid cysteine at codon 1012 to alanine, which results in a premature stop codon 109 amino acids downstream. This variant is predicted to result in a loss of function of the ADAMTS13 protein. To date, this variant has not been reported in the literature in patients with severe congenital ADAMTS13 deficiency, however, different frameshift variants are a known cause of ADAMTS13 deficiency [17]. In summary, the collective evidence supports ADAMTS13 c.3033delC, p.Cys1012AlafsX109 as a pathogenic variant in regards to severe congenital ADAMTS13 deficiency.

This case was previously presented as a meeting abstract. (Abstract: 64th Annual Scientific and Standardization Committee Meeting of International Society of Thrombosis & Hemostasis, July 18-21, 2018).

## Conclusions

In conclusion, this case of congenital TTP exhibited a homozygous ADAMTS13 frameshift variant. The clinical course of this patient highlights the diagnostic difficulties encountered as well as signifies the need for timely diagnosis and treatment of congenital TTP. Therefore, it is imperative to consider congenital TTP in the differential diagnosis of any neonate presenting with hyperbilirubinemia and thrombocytopenia.
